# Diagnostic Value of Hepatic Vein Ultrasound in Early Detection of Liver Cirrhosis

**DOI:** 10.22086/gmj.v0i0.1140

**Published:** 2018-08-11

**Authors:** Hadi Zare Marzouni, Behrooz Davachi, Mahdi Rezazadeh, Mahmoud Salehi Milani, Sedighe Matinfard

**Affiliations:** ^1^Department of Immunology, Faculty of Medicine, Mashhad University of Medical Sciences, Mashhad, Iran; ^2^Student Research Committee, Faculty of Medicine, Mashhad University of Medical Sciences, Mashhad, Iran; ^3^Department of Radiology, Faculty of Medicine, Mashhad University of Medical Sciences, Mashhad, Iran

**Keywords:** Liver Cirrhosis, Hepatic Vein, Liver, Sonography

## Abstract

**Background:**

Cirrhosis is a common disease that destroys liver cells, and it has various etiologies. The early diagnosis of cirrhosis can be effective in improving prognosis. Considering the availability and affordability of ultrasound devices throughout the world, in this study we evaluated the diagnostic value of hepatic vein ultrasound examination in the early detection of liver cirrhosis.

**Materials and Methods:**

In this study, 45 patients referred to the radiology department of Ghaem Hospital for liver biopsy were evaluated for changes in the hepatic veins under ultrasound with a transducer of 5-7MHz. One piece of the hepatic vein was selected for ultrasound, and the wall of hepatic veins was examined for straightness and uniformity of echogenicity. Subsequently, patients underwent biopsy by ultrasound guide. Of all the study subjects, eight patients presented mild chronic hepatitis without fibrosis, four patients were diagnosed with fatty liver, and 33 patients had severe cirrhosis or chronic hepatitis with fibrosis.

**Results:**

Our results showed that hepatitis patients with or without fibrosis had irregular and wavy hepatic vein wall (impairment in straightness) with non-uniform echo (impairment in uniformity of echogenicity). While patients without hepatitis and cirrhosis, but diagnosed with fatty liver, had a smooth and regular hepatic vein wall with a uniform echo.

**Conclusion:**

The present study showed that hepatic vein examination in terms of echogenicity and straightness could be used to diagnose hepatitis, its severity as well as its course towards fibrosis and cirrhosis.

## Introduction

Liver cirrhosis is a common disease that results in damage to liver cells. It has diverse etiologies that result in fibrosis and regenerative nodules [1, 2]. Although the mortality rate of patients with this disease has decreased by about 30% in recent years, due to the vital role of the liver, the cirrhosis of cells is still one of the most common causes of morbidity [1, 3, 4]. The causative agents include alcohol abuse, chronic infections, hepatitis, autoimmunity, biliary disease, hemochromatosis, Wilson’s disease, a1 antitrypsin (A1-AT)

deficiency, and cystic fibrosis. The complications associated with liver cirrhosis include hypertension and its outcomes, ascites, liver encephalopathy, gastric variceal hemorrhage, hepatorenal syndrome, splenomegaly, spontaneous bacterial peritonitis and hepatoma [5-8].

Patients with liver cirrhosis undergo repeated ultrasound examinations to observe parenchymal abnormalities, hepatic vascular examination, hypertensive portal manifestations and screening for hepatocellular carcinoma. Advanced ultrasound manifestations of cirrhosis include a nodular lining of the shrunken liver, reduced blood flow to the port vein and dilated intrahepatic arteries [9, 10]. In most patients with cirrhosis, diagnosis occurs only in the late stages of the disease when hepatic failure develops. For this reason, only protective therapeutic measures are taken for these patients [11].

Since early diagnosis of cirrhosis can effectively help improve disease prognosis, researchers are always looking for effective ways to diagnose the disease at early stages [12]. Also, studies have shown that by eliminating the causative agent of cirrhosis, there is still the risk of relapse. This case has been observed mostly in the successful treatment of chronic hepatitis C [13]. Moreover, fibrosis in patients with alcoholic liver was improved by abstaining from alcohol [14].

Some studies suggested that cirrhosis could cause changes in waveforms of vein ports, liver veins and liver arteries [15, 16].

It is likely that the identification of changes on the surface of liver veins can be effective in the early diagnosis of hepatic cirrhosis [17].

Since ultrasound is an accessible and inexpensive modality in the whole world, it is possible to identify the changes in liver veins in ultrasound [18].

If ultrasound examination of liver veins and identification of the association of liver changes in early diagnosis of hepatic cirrhosis is proven, this method can be a suitable method for the early diagnosis and screening of the disease [18, 9].

Therefore, according to the stated content, this study was conducted to evaluate the diagnostic value of liver vein ultrasound in the early diagnosis of hepatic cirrhosis.

## Materials and Methods

The sonographic examination of hepatic veins is a predictable method in all liver sonographic analyses at our organization. The Ethics Committee approval of Mashhad University of Medical Sciences was obtained for this study. Also, this study was registered at Iranian Registry of Clinical Trials (code: IR.MUMS.REC.1389.93).

### Patients

The participants of this experimental study included all patients who were referred by a gastroenterologist to the radiology department of Ghaem Hospital, Mashhad University of Medical Sciences, Iran during a study in 2016, based on suspicion of liver cirrhosis, for liver biopsy. The sample size was calculated to be 41 patients based on the test of proportion in the society, according to the frequency of positive cases of liver vein ultrasound in patients with cirrhosis (70%); and d=1.5p α=0.05, and considering 10% reduction in sample size, 45 people were considered. Patients whose pathology result was unavailable were excluded.

### Study Design

First, an informed written consent was

obtained from all patients with hepatic dysfunction, who were candidates for liver biopsy. Then, for measuring changes in

hepatic veins, the participants underwent ultrasound with My lab-60 using a transducer of 2-5MHz. The right hepatic vein and its branches are more suitable for examining the echo of the vein wall; thus, to have a better view, a peripheral segment of the hepatic vein that was perpendicular to the transducer was selected for ultrasound evaluation.

The venous walls were assessed for straightness and echogenicity. The images were taken deep and with the most appropriate magnification.

The venous segment, which had a length of at least 15mm and a diameter of 3mm, was selected for ultrasound. Thereafter, the patients underwent biopsy by ultrasound guide.

Three pathology slides were prepared from each sample and were identified by a pathologist.

The ultrasound results of the patients in these two groups were matched with pathological findings, and the results were evaluated.

### Straightness of Hepatic Vein Wall Procedure

**Figure-1:**
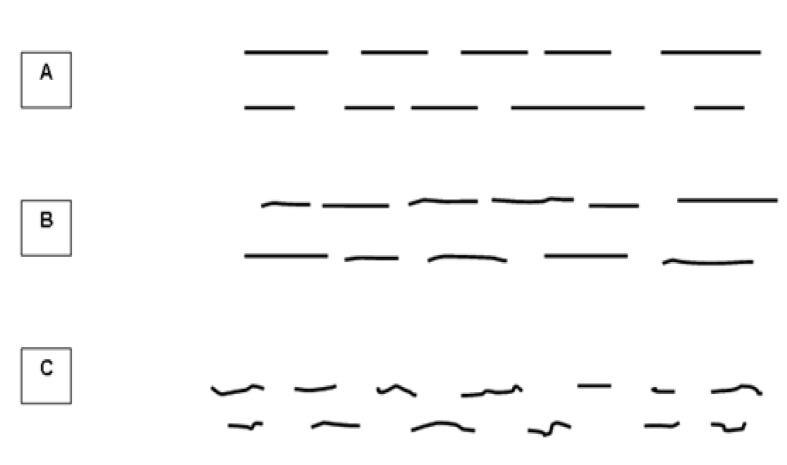
Schematic diagram is showing hepatic vein straightness. A: Straight; B: Slightly wavy; C: Very wavy

**Figure-2:**
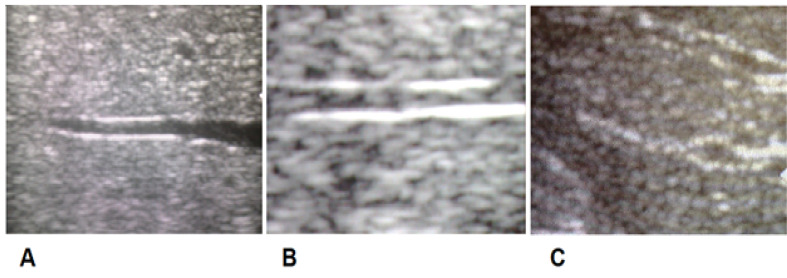
Sonograms showing hepatic vein straightness. A: The hepatic wall is normal and straight (type A). B: The hepatic wall is slightly wavy (type B). C: The hepatic wall is very wavy (type C)

The straightness of the hepatic vein wall was divided into three types: A, B, and C (Figures-1 and 2). Type A (Figure-2A) indicated a pattern of the straight wall and can be seen in normal livers without any alteration. Type B (Figure-2B) had a mild grade of irregularity producing a “slightly wavy” pattern. Type C (Figure-2C) explained an increasingly distorted design of the wall with a clear “very wavy” pattern. Both types B and C indicated alteration of the normal straight morphological characteristic.

### Uniformity of the Hepatic Vein Wall Echogenicity Procedure

**Figure-3:**
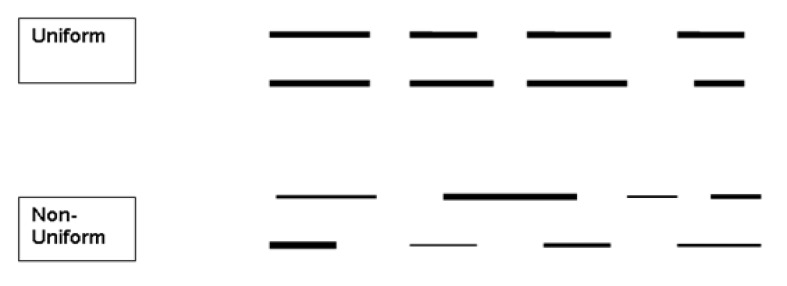
Schematic diagram is showing uniformity of hepatic vein wall echogenicity: uniform morphology and non-uniform

**Figure-4:**
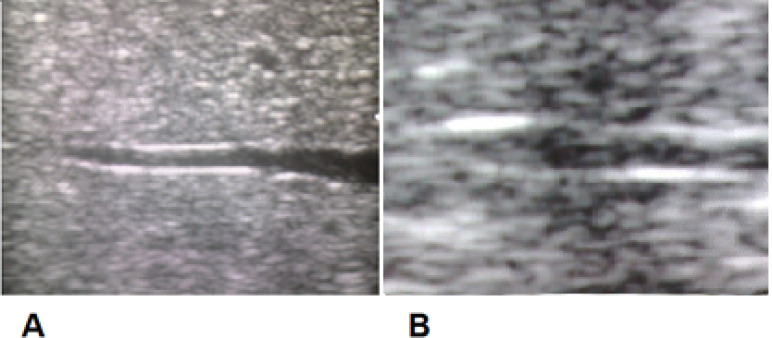
Sonograms showing hepatic vein uniformity of wall echogenicity. A: Uniform hepatic vein wall. B: Non-uniform hepatic vein wall

In liver cirrhosis, the increase of hepatic vein wall nodularity was envisaged to alter the wall, generating unevenness and floating echogenicity when imaged vertically to the ray (Figures-3 and 4). A normal hepatic vein wall could be envisaged to have a uniform echogenicity and brightness throughout a 15-mm segment. An unfailing hepatic vein wall echogenicity was indicated as “uniform” (Figures-3 and 4A), and the nonexistence of it was assumed to be “non-uniform” (Figures-3 and 4B).

### Hepatic Pathology Procedure

**Figure-5:**
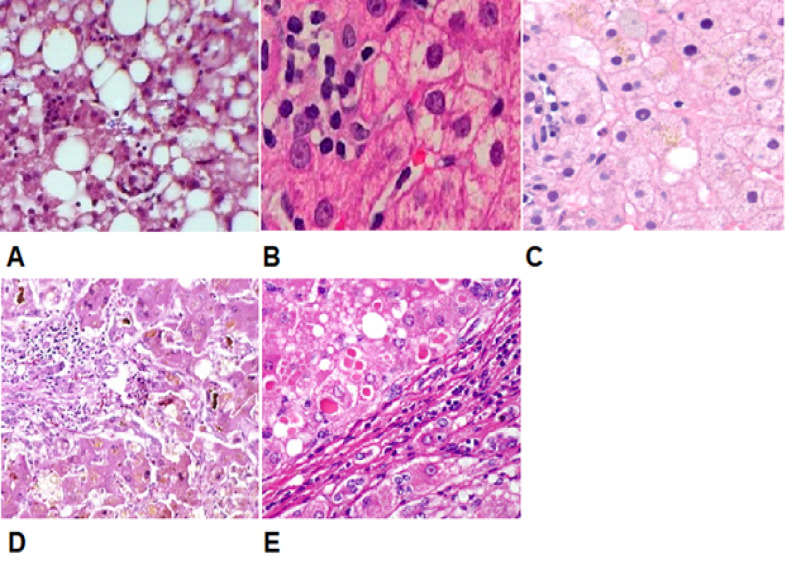
The pathology finding in samples in 5 groups. A: liver fatty disease; B: Mild chronic hepatitis; C: Mild fibrosis symptoms in addition to mild chronic hepatitis; D: Severe chronic hepatitis plus; E: Severe cirrhosis (the samples stained by H&E staining and magnification was 100X)

After measuring changes in hepatic veins by ultrasound, a biopsy was conducted for the patients by using an ultrasound guide. Three pathology slides were prepared from each sample by Hematoxylin and eosin (H&E) staining and were identified by a pathologist. After obtaining the pathological results of the samples, patients had five types of pathological liver damage: 1) liver fatty disease, 2) mild chronic hepatitis, 3) mild fibrosis symptoms in addition to mild chronic hepatitis, 4) severe chronic hepatitis plus fibrosis, and 5) severe cirrhosis (Figure-5). Finally, the patients were divided into two groups: cirrhotic and non-cirrhotic. The ultrasound results of the patients in these two groups were matched with the pathological findings, and the results were evaluated.

### Data Analysis

The results were analyzed using SPSS Version 24 software. Descriptive data were composed of the frequency distribution table, central indexes, distribution, and percentages. Continuous quantitative data were compared between the different groups using the independent t-test, ANOVA and Chi-Square test. A P-value <0.05 was considered as the level of significance.

## Results

In this study, 45 patients with hepatic problems, who were referred for liver biopsy were evaluated. Of all the studied samples, 29 patients (64.4%) were male, and 16 patients (35.6%) were female. The mean±SD age of the samples was 43.52±9.89 years. Of the samples, 18 (40%) were smokers. Also, five people (11.11%) used alcohol (Table-1).

**Table-1:** The Demographic Characteristics in Samples

**Variation**		**LFD** **(n=4)**	**MCH** **(n=8)**	**MFS & MCH** **(n=17)**	**SCH+ Fibrosis (n=4)**	**SC** **(n=12)**	**Total** **(n=45)**	**P-value**
**Sex**	Male	3(75%)	5(62.5%)	10(58.82%)	3(75%)	8(66.66%)	29(64.4%)	0.351
	Female	1(25%)	3(37.5%)	7(41.18%)	1(25%)	4(33.34%)	16(35.6%)	
**Age**		41.23±9.71	44.8±5.21	43.08±13.02	47.28±10.36	42.81±8.51	43.52±9.89	0.061
**BMI**		25.27±3.35	23.24±2.51	26.61±2.99	25.25±4.28	25.42±3.49	25.45±3.18	0.184
**Smoking**		2 (50%)	0	5(29.41%)	3(75%)	8(66.66%)	18(40%)	0.16
**Alcohol**		1(25%)	1(12.5%)	1(5.88%)	0	2(16.66)	5(11.11%)	0.24

**LFD:** Liver fatty disease; **MCH:** Mild chronic hepatitis; **MFS & MCH:** Mild fibrosis symptoms in addition to mild chronic hepatitis; **SCH:** Severe chronic hepatitis plus; **SC:** Severe cirrhosis

In the pathological examination, patients had five types of pathological liver damage; four patients (8.88%) had the only liver fatty disease, while eight patients (17.77%) had mild chronic hepatitis. In addition, 17 patients (37.77%) had mild fibrosis symptoms in addition to mild chronic hepatitis. Four patients (8.88%) had severe chronic hepatitis plus fibrosis while 12 patients (26.66%) had severe cirrhosis.

Overall, 33 patients (73.33%) had symptoms of cirrhosis due to the presence or absence of fibrosis, and 12 patients (26.64%) had no histopathological symptoms of cirrhosis.

The results of ultrasound examination of hepatic vein showed that in terms of hepatic vein wall straightness, four patients (8.88%) had a smooth and regular vein wall and the hepatic vein of the remaining patients (91.11%) had an irregular and wavy wall.

Furthermore, in terms of uniformity of hepatic vein echogenicity examination, 12 patients (26.64%) had even and smooth vein wall, while 33 patients (73.33%) had non-uniform vein walls with heterogeneous echogenicity. All 33 patients with severe cirrhosis, severe chronic hepatitis with fibrosis and mild chronic hepatitis in the pathology diagnosis, had fibrosis with the non-uniform hepatic vein. Moreover, all 33 patients with cirrhosis of the liver had an irregular and wavy hepatic vein wall in terms of hepatic vein wall straightness.

Of the 12 patients without histopathological symptoms of cirrhosis, four patients (33%) had smooth, regular and normal hepatic vein wall, who had no histopathological signs of hepatitis and those who had fatty liver. Eight patients (67%) had an irregular and wavy wall that showed signs of severe chronic hepatitis in the pathology. In fact, patients with hepatitis C or without cirrhosis had non-uniform hepatic vein walls whereas patients without hepatitis and cirrhosis with a fatty liver had normal, smooth and regular hepatic vein walls.

Twelve patients with mild chronic hepatitis lacking fibrosis and fatty liver, who had no symptoms of fibrosis and cirrhosis, had a hepatic vein wall with a uniform echo and uniform thickness.

## Discussion

Liver biopsy is still regarded as the gold standard for the diagnosis of liver cirrhosis, but it has problems such as causing complications and technical problems in sample collection [19, 20]. Different ultrasound parameters have been used to diagnose cirrhosis [1, 9]. These parameters mainly depend on hepatic echogenicity and morphological changes but have limited sensitivity [1, 9, 16].

Radiologic evaluation plays a vital role in the identification and follow-up of patients with liver cancer, and ultrasound has a floating accuracy of 76-93%. Methods such as hepatic transit time measurement, liver elastography, and magnetic resonance imaging spectroscopy have not been used routinely [21-23]. Several ultrasound parameters such as morphological and vascular changes have been used to diagnose cirrhosis, most of which were non-sensitive and non-specific [12, 24].

However, evaluation of nodularity of the liver surface has a high sensitivity and specificity in the diagnosis of hepatic cirrhosis [25]. Identification of liver nodularity in patients with ascites was clearer with a fluid-organ surface [26, 27]. Our study hypothesized that the blood (fluid) in the vein provides an appropriate level of fluid interactions between the liver and its parenchyma for evaluating the liver nodularity. Therefore, this study aimed to evaluate the diagnostic value of hepatic vein ultrasound in the early detection of liver cirrhosis.

In the present study, hepatic vein wall straightness and the uniformity of echogenicity were investigated, and in both cases, a high correlation was observed between liver cirrhosis and abnormalities in the shape and echo of the vein wall. All studied cases of cirrhosis, including severe cirrhosis or chronic hepatitis with fibrosis presented irregular, wavy, and non-uniform hepatic vein walls. Also, patients with fatty liver without symptoms of hepatitis or cirrhosis in the pathology had smooth, regular and even vein wall. Furthermore, in assessing the hepatic vein walls of the patients, it was found that even chronic mild hepatitis without fibrosis and cirrhosis could disturb the straightness of liver veins and make it wavy. However, in this case, the hepatic vein wall was uniform, and its thickness was also uniform. Finally, the results of this study showed that patients presenting hepatitis with or without fibrosis had an irregular hepatic vein wall (disorder in straightness) with non-uniform echo (disorder in echogenicity) while patients without hepatitis and cirrhosis presenting fatty liver had a regular and smooth hepatic vein wall.

Our results are similar to those of Vessal et al., and suggest that liver vein examination for uniformity and straightness could be used to diagnose hepatitis, its severity and its course towards fibrosis and cirrhosis. In their study, the liver vein morphology was evaluated as a marker for cirrhosis, suggesting that the use of hepatic vein for nodularity was more useful than an examination of the live surface [18].

The major restrictions and areas for further study shown by our study include the number of patients analyzed. It is important to emphasize that this was a pilot study requiring further assessment and validation with more patients presenting liver cirrhosis of diverse etiologies as well as a spectrum of patients with steatosis and fibrosis. It is essential to diagnose the early stages of the liver cirrhotic subtypes.

To substantiate the above suggestion, examination of a larger sample size of patients with cirrhosis is recommended due to various causes such as cirrhosis due to alcohol intoxication, cirrhosis following hemochromatosis and Wilson, biliary cirrhosis, etc.

## Conclusion

Finally, the present study showed that examination of changes in the hepatic vein in terms of hepatic vein wall straightness and uniformity of echogenicity could be used as a valuable and important novel parameter in the evaluation of patients with liver cirrhosis. Also, it can be used for the diagnosis of hepatitis, its severity and its progression towards fibrosis and cirrhosis. This method is an ordinary, susceptible parameter, is reproducible and a valuable excess to the sonographic armamentarium in the recognition and follow-up of patients with liver cirrhosis.

## Conﬂicts of Interest

The author(s) indicated no conﬂicts of interest.
